# UV Sensor Based on Fiber Bragg Grating Covered with Graphene Oxide Embedded in Composite Materials

**DOI:** 10.3390/s20195468

**Published:** 2020-09-24

**Authors:** Piotr Lesiak, Karolina Bednarska, Krzysztof Małkowski, Łukasz Kozłowski, Anna Wróblewska, Piotr Sobotka, Kamil Dydek, Anna Boczkowska, Tomasz Osuch, Alicja Anuszkiewicz, Wojciech Lewoczko-Adamczyk, Henning Schröder, Tomasz Ryszard Woliński

**Affiliations:** 1 Faculty of Physics, Warsaw University of Technology, 00-662 Warszawa, Poland; karolina.bednarska.dokt@pw.edu.pl (K.B.); krzys.malkowski@gmail.com (K.M.); lukasz.kozlowski4.stud@pw.edu.pl (Ł.K.); anna.wroblewska@pw.edu.pl (A.W.); piotr.sobotka@pw.edu.pl (P.S.); tomasz.wolinski@pw.edu.pl (T.R.W.); 2 Centre for Advanced Materials and Technologies CEZAMAT, 02-822 Warszawa, Poland; 3 Faculty of Materials Science and Engineering, Warsaw University of Technology, 02-507 Warszawa, Poland; kamil.dydek@pw.edu.pl (K.D.); anna.boczkowska@pw.edu.pl (A.B.); 4 Faculty of Electronics and Information Technology, Institute of Electronic Systems, Warsaw University of Technology, 00-665 Warszawa, Poland; T.Osuch@elka.pw.edu.pl (T.O.); a.anuszkiewicz@elka.pw.edu.pl (A.A.); 5 Abt. SIIT/Optical Interconnection Technology, Fraunhofer-Institut für Zuverlässigkeit und Mikrointegration (IZM), 13355 Berlin, Germany; Wojciech.Lewoczko-Adamczyk@izm.fraunhofer.de (W.L.-A.); Henning.Schroeder@izm.fraunhofer.de (H.S.)

**Keywords:** composites, fiber Bragg grating sensor, UV radiation, fiber optic sensor

## Abstract

Polymer–matrix composites degrade under the influence of UV radiation in the range of the 290–400 nm band. The degradation of polymer–matrix composites exposed to UV radiation is characterized by extensive aging of the epoxy matrix, resulting in deterioration of their mechanical properties. Glass fibers/epoxy resin composites were made by an out-of-autoclave method whereas a fiber optic sensor was placed between different layers of laminates. In our work, we used a fiber Bragg grating sensor covered with graphene oxide and embedded in a polymer matrix composite to monitor UV radiation intensity. Measurements of UV radiation may allow monitoring the aging process of individual components of the polymer composite. In order to estimate the number of microcracks of epoxy resin, microstructure observations were carried out using a scanning electron microscope.

## 1. Introduction

Due to their very good mechanical properties, epoxy resins are very often used as a matrix of composites or coatings in a variety of industrial applications: automotive, energy or construction, electromagnetic, electric and electromechanical, particularly in concrete constructions. Such nanocomposites should possess good adhesion with cement/concrete, as well as high fire resistance and good weatherability against UV, alkaline and saline environments [1,2,3]. 

Chemical and physical changes of structures made of epoxy resins occur through the action of atmospheric conditions such as sunlight, oxidation, and other external factors [4,5,6]. The reason for the absorption of UV radiation by epoxy resin and as a result its degradation is the presence of an aromatic moiety in their structure. UV radiation, which has influence on discoloration and chalking of epoxy surface, is the major reason for limiting the use of epoxies for outdoor applications [7,8].

Previous studies have confirmed the negative effect of UV radiation on epoxy matrix composites. However, not all the types of UV radiation can reach the composite material exposed to sunlight because the ozone layer absorbs 100% of UVC radiation (100–280 nm) and majority of UVB radiation (280–315 nm). Only UVA radiation (315–400 nm) and a small part of UVB radiation reach the Earth’s surface and adversely affect epoxy resin [9]. Yan, Chouw and Jayaraman have studied the combined effect of ultraviolet (UV) radiation and water spraying on the mechanical properties of a flax fabric reinforced epoxy composite [10]. The aim of their work was to study the durability of such composites used in civil engineering. The research conducted by the authors confirmed that UV radiation and water spraying had an impact on degradation of epoxy matrix composites resulting in discoloration, matrix erosion, microcracking and voids formation. Other studies confirm the negative effect of UV radiation on epoxy composites, which resulted in darkening of the surface and formation of microcracks in epoxy resin/nanoclay composites [1]. The effect of UV radiation on carbon fiber reinforced polymers (CFRP) was also investigated and, as in the above studies, epoxy matrix degradation was also observed, which resulted in deterioration of the mechanical properties of composites. In that case, microcracks, matrix erosion, and fiber debonding were also observed [11].

Methods to counteract epoxy resin degradation can be found in the literature. By introducing antioxidants and photo stabilizers, which include titanium dioxide (TiO_2_), and zinc oxide (ZnO), high-energy radiation can be attenuated or suppressed [7]. Carbon black (CB) is another additive used to stabilize polymers against UV radiation. The effectiveness of application depends on the type and size of CB particles as well as the degree of dispersion and concentration of the filler in the polymer matrix [12,13]. 

Interesting possibilities to monitor the aging processes of the epoxy matrix are provided by the use of fiber optic sensors laminated in a composite material [14]. Fiber optic sensors have many advantages over conventional sensors [15], such as small size, electrical safety or immunity to electromagnetic interference. Solutions are known to monitor the distribution of stress or temperature [16,17], however the measurement of UV radiation using fiber optic sensors has not been reported. In this manuscript we propose the use of a laminated fiber optic sensor with a graphene oxide (GO) coated fiber Bragg grating (FBG) for UV measurement inside the composite material. A general idea of this sensor assumes that a temperature change around the fiber can be induced by UV radiation as shown in our previous publication [18]. The absorbance spectrum of an aqueous solution of GO typically peaks at 230 nm while visible and infrared radiation is not significantly absorbed. In laminated FBG sensors, scattered UV light increases the internal energy of GO and consequently locally raises the surface temperature of optical fiber [19]. Consequently, temperature changes modify stress distribution within the fiber and result in a change in Bragg’s wavelength [20].

## 2. Materials and Methods 

Typically, the refractive index variation in an FBG changes sinusoidally along the grating length [21,22]. The wavelength λ_B_ which is reflected due to these periodic changes is called Bragg wavelength and is expressed by the following equation:

(1) $$\lambda_{B}=2n_{eff}\Lambda$$

with $n_{eff}$ being the effective refractive index of propagating core mode and Λ the grating period, i.e., a distance between neighboring maxima in periodically induced refractive index changes in the fiber core.

Any temperature change in the vicinity of the FBG results in a Bragg wavelength shift. This shift is due to two effects: thermal expansion (which changes the period of the FBG) and thermo-optic effect (stemming from a change in the effective refractive index in the optical fiber). In the case of silica optical fibers, the latter is predominant. Formula (2) describes the influence of the change of temperature ΔT on the Bragg wavelength [23]:

(2) $$\Delta \lambda_{B}=2\left(\Lambda \frac{\partial n_{eff}}{\partial T}+n_{eff}\frac{\partial \Lambda }{\partial T}\right)\Delta T$$

A typical temperature sensitivity of the FBG within the 1550 nm wavelength range is c.a. 10 pm/K.

FBGs were written by a krypton fluoride (KrF) excimer laser manufactured by Coherent using the phase mask technique. This laser generates 15 ns pulses at a wavelength of 248 nm. During the gratings inscription, 3 mJ energy and 500 Hz repetition rate of laser pulses were set. Before the inscription of the grating, an SMF-28 standard single-mode optical fiber manufactured by Corning was high-pressure hydrogen loaded at room temperature to enhance its photosensitivity. During the grating inscription, 3 mJ laser pulse energy and 500 Hz repetition rate were set, while the process of FBG formation was monitored on-line to obtain similar spectral responses for all in-written gratings.

**Figure 1:**
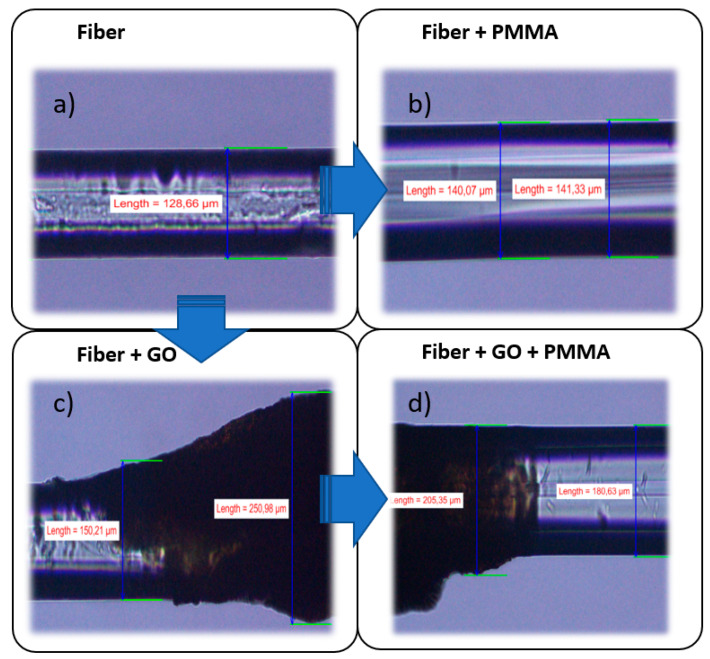
Images of optical fiber without any protective coating (**a**), partially covered by poly(methyl methacrylate) (PMMA) (**b**), partially covered by graphene oxide (GO) film (**c**), optical fiber and GO film covered by PMMA (**d**) observed in an optical microscope (magnification 20×).

Thin graphene oxide films (manufactured by Graphene Supermarket) were prepared by the vacuum filtration method. GO suspension with a concentration of 0.1 mg/mL was used in the filtration process. The suspension was filtered onto a mixed cellulose ester membrane filter (MCE, 0.45 μm pore-size, 25 mm diameter). The formed layer on the filter was left under air flow to complete drying. Next, the dry GO film was cut and immersed in an acetone bath to dissolve the filter. After removing the filter residue, the clean GO layer was transferred on a selected optical fiber and gently dried with a nitrogen stream. The thickness of the GO film was about 100 nm (measured by atomic force microscopy). Unfortunately, when applying the GO layer to the optical fiber, the layer tends to surface irregularly and sticks. Therefore, in microscopic images (Figure 1c,d), GO creates layers that appear thicker than 100 μm.

Since the GO layer located on the surface of the optical fiber is very fragile, that is why it should be properly protected against lamination in a composite material. Poly(methyl methacrylate) (PMMA) was used as a protective material for the GO layer [24], as it exhibits good adhesion with both the silica glass from which the optical fiber is made (Figure 1b) and GO (Figure 1d). In addition, it protects the GO layer from reduction. The absorption of PMMA peaks for wavelengths below 300 nm [25]. Since the wavelength of the laser we used in experiments was 404 nm, the PMMA layer did not contribute to the temperature change. Additionally, UV radiation for wavelengths below 280 nm is negligible (absorption in the atmosphere), therefore PMMA does not affect the sensor in any way because the sensor’s range of interest is between 300 and 400 nm. 

**Figure 2:**
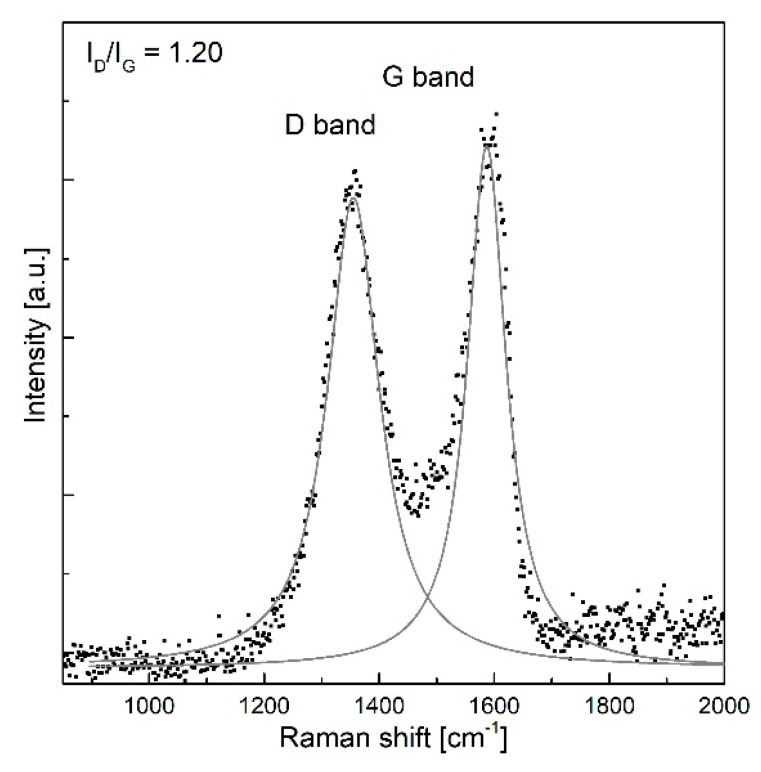
Raman spectrum of the GO thin film. Intensity ratio and peak positions were obtained from Lorentzian fit.

Figure 2 presents the Raman spectrum of the thin GO film deposited on the surface of the optical fiber. Two main bands were observed in the spectrum: D (1345.9 cm^−1^) and G (1590.5 cm^−1^), in which peak positions and intensity of each band were obtained from Lorentzian fitting. Based on a relative intensity ratio of the D and G modes (I_D_/I_G_ = 1.20) we confirmed the purity and presence of GO on the optical fiber before covering with a thin layer of PMMA [26]. The Raman spectra were collected by using a 514 nm laser in a Renishaw inVia spectrometer in ambient conditions. Measurements were performed for low laser power (below 0.3 mW) to avoid the sample heating and reduction processes. 

**Figure 3:**
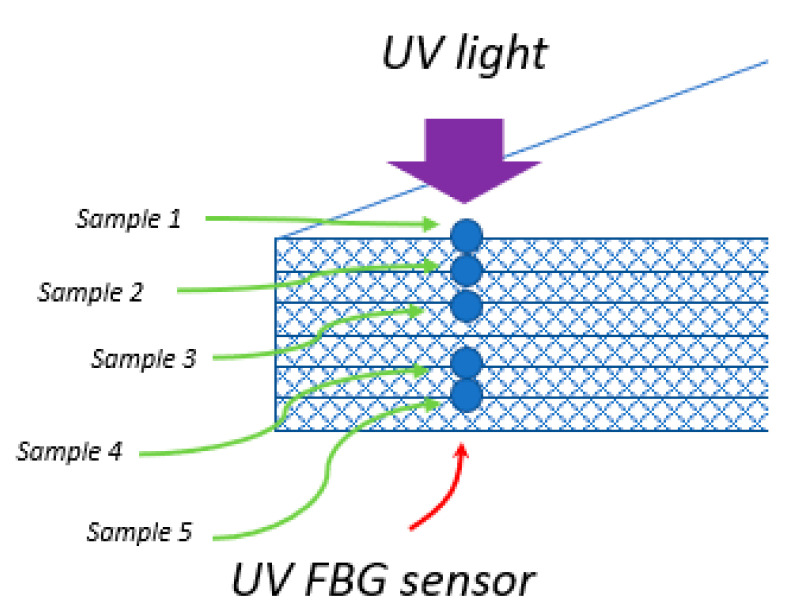
Schematic of UV sensors arrangement in a composite sample.

**Figure 4:**
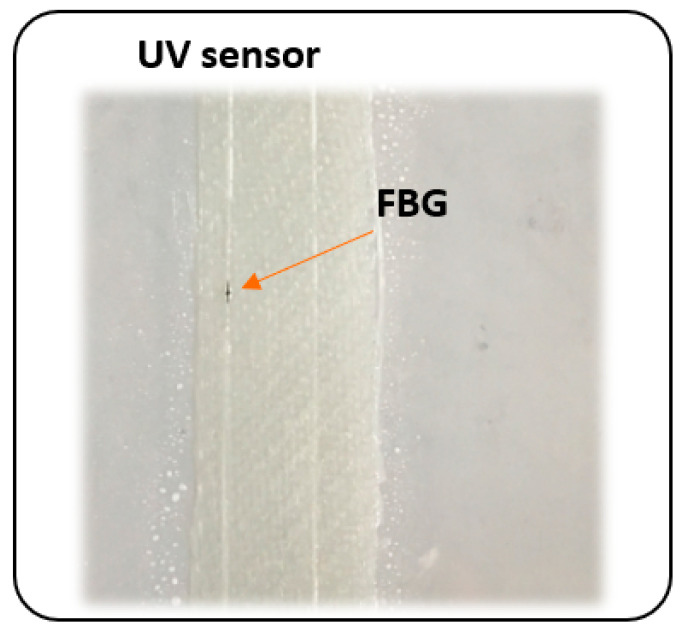
The image of FBG UV sensor arrangement in a composite sample.

In order to verify the performance of a UV sensor located at a different distance from the surface of composite, three types of laminates were made using out-of-autoclave method, formed by hand lay-up. In all cases, epoxy resin with a trade name Epidian52, supplied by Sarzyna S.A. mixed with a triethylenetetramine hardener (Z1) in a ratio 100:13 was used as a polymer matrix, while Interglas 92125 glass fabric supplied by Interglas (280g/m2, twill 2/2 weave) was used as reinforcement. Laminates were made using a glass plate as a base, from which they were separated with a release film. No chemical release-agent was used. The depth of introduction of the FBG based UV sensor was different in all laminates—in the first case it was laid on the surface, in the second between the first and second layers, and in the third laminate—between the second and third one (Figure 3). In addition, a reference FBG sensor without GO coating was placed in each sample (Figure 4). All laminates were cured for 24 h at room temperature.

**Figure 5:**
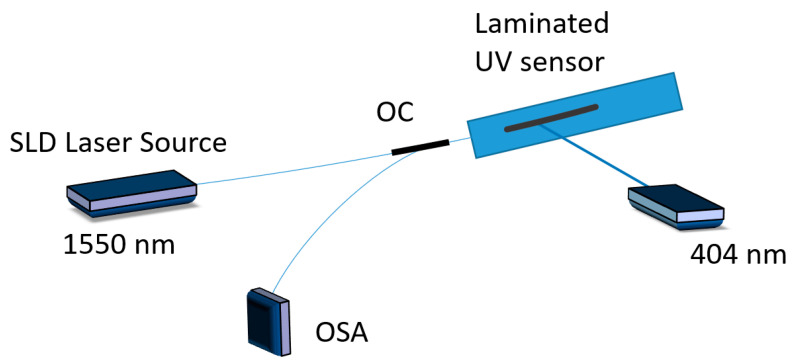
Experimental setup.

The experiment was conducted as follows: light from a superluminescent diode (SLD) source with a central wavelength of 1550 nm and a spectral width of 100 nm was launched into the fiber. The Bragg wavelength reflected from the FBG was redirected by the circulator through its third port into the optical spectrum analyzer (OSA). A 404 nm ‘heating’ laser beam was focused on the GO covered part of the fiber where the FBG was inscribed (Figure 5). The size of the beam in focus was 2 × 3 mm and it was positioned in a way that only 3 mm long central section of the FBG was illuminated.

## 3. Results and Discussion

**Figure 6:**
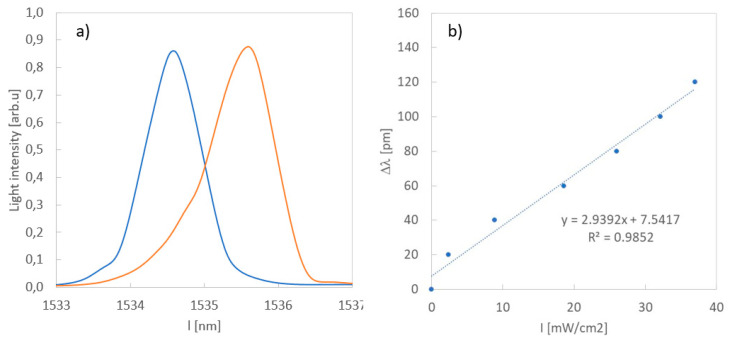
Bragg wavelengths of the fiber Bragg grating (FBG) sensor covered by PMMA before (blue) and after (orange) illumination by the 404 nm laser light with intensity 500 mW/cm^2^ (**a**) and dependence of the Bragg wavelength change on the intensity of UV light in the same sensor (**b**).

Initially the FBG sensor was investigated prior to lamination in the composite material. For this purpose, FBG covered with GO and a protective layer was placed in the measurement system presented in Figure 5. The Bragg wavelength changed by 1.1 nm when the UV sensor was illuminated by the 404 nm laser light with intensity equal to 500 mW/cm^2^ (Figure 6a). An accurate measurement of the dependence of the Bragg wavelength change on the intensity of light illuminating the sensor (inset in Figure 6) yields sensor sensitivity equal to 3 pm/mW/cm^2^ (Figure 6b). 

In addition, it was noticed that thanks to the PMMA protective layer, the UV sensor could safely operate for a greater range of light intensity changes. Without the protective layer, the safe level of light intensity for which the GO layer was not damaged was 160 mW/cm^2^ (Table 1). After coating GO with the PMMA protective layer, the light intensity range increased to 500 mW/cm^2^.

**Figure 7:**
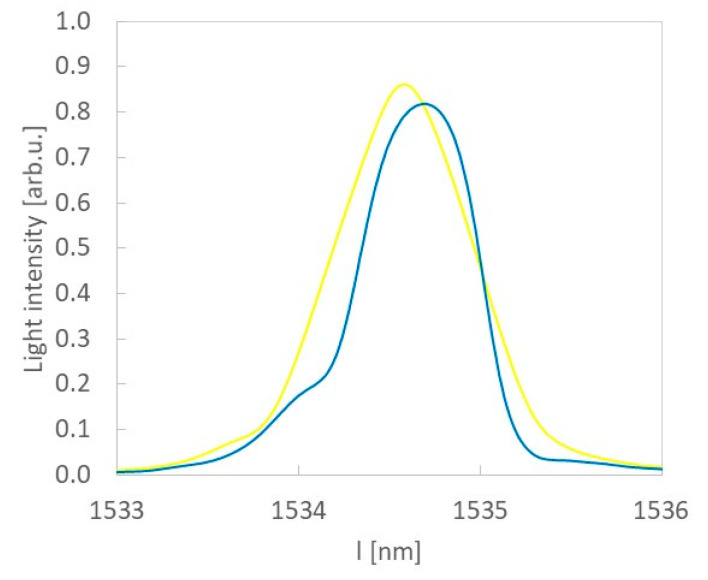
Bragg wavelengths before (yellow) and after (blue) of lamination process FBG sensor in sample 3.

As explained in [27], the Bragg wavelength slightly changes after lamination in the composite material. This is the effect of technological shrinkage that arises in the composite material as a result of the lamination process. The impact of technological shrinkage can be minimized by adding a protective layer [28]. In our case it is PMMA, however, the thickness of the protective layer turned out to be insufficient and a residual Bragg wavelength shift of 0,1nm was observed (Figure 7). In the following measurements, the Bragg wavelength after lamination was taken as the reference value.

**Figure 8:**
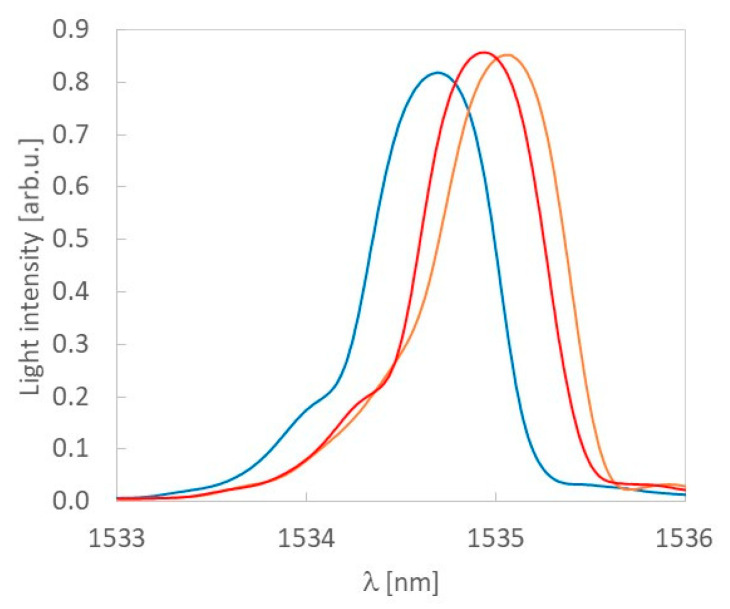
The Bragg wavelength of a UV sensor before (blue) and after illumination by the 404 nm laser light with intensity 500 mW/cm^2^ for FBG sensors laminated in the sample 3 (orange) and the sample 4 (red).

The Bragg wavelength of the UV sensors laminated in the sample 3 and the sample 4 illuminated by 404 nm laser light with intensity 500 mW/cm^2^ is presented in Figure 8. The difference between the Bragg before the UV illumination for both samples was small, therefore these two samples were selected for comparison, and for greater clarity, one spectrum was used before irradiating the samples with UV. A change in the Bragg wavelength is equal to 0.35 nm and 0.23 nm for sensors laminated in samples 3 and 4, respectively. The change decreases with the depth of FBG lamination but the sensitivity of the laminated sensor is constant. Moreover, within the scope of its action this change results directly from the light scattering in the composite material. 

Light passing through the composite material is strongly dispersed due to its large heterogeneity [29]. This heterogeneity results from the fact that the composite material consists of two components: resin and reinforcement. In addition, the laminates were made using the out-of-autoclave method, which is why the composite material used in our experiment is characterized by very high dispersion. In general, the absorption can be expressed by the following equation [30]:

(3) $$I=I_{0}e^{-\alpha d}$$

where *α* is the attenuation coefficient, *d* is the sample thickness, *I* and *I*_0_ are output and input light intensity, respectively. 

**Figure 9:**
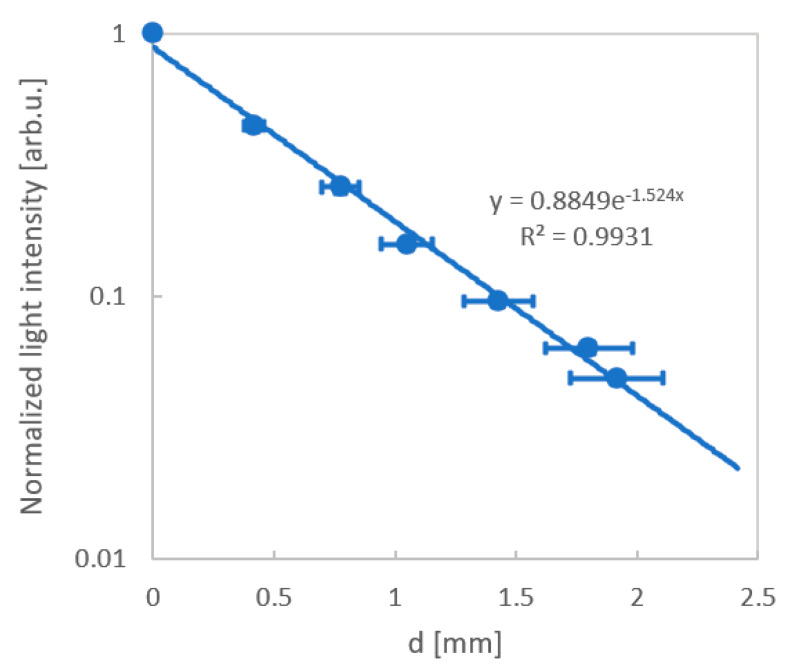
Dependence of light transmission on the thickness of the composite material.

The measurement of the effect of thickness on light transmission was made using composite materials with varying thicknesses, from 0.4 to 2.0 mm. This allowed to determine the attenuation factor α = 1.52 mm^−1^ (Figure 9). 

Both light scattering as well as absorption result in less light reaching the UV-laminated sensor in the composite material in comparison with the non-laminated sensor. The reduction in the Bragg wavelength change for UV sensors laminated at different depths in the composite material confirms this effect.

**Figure 10:**
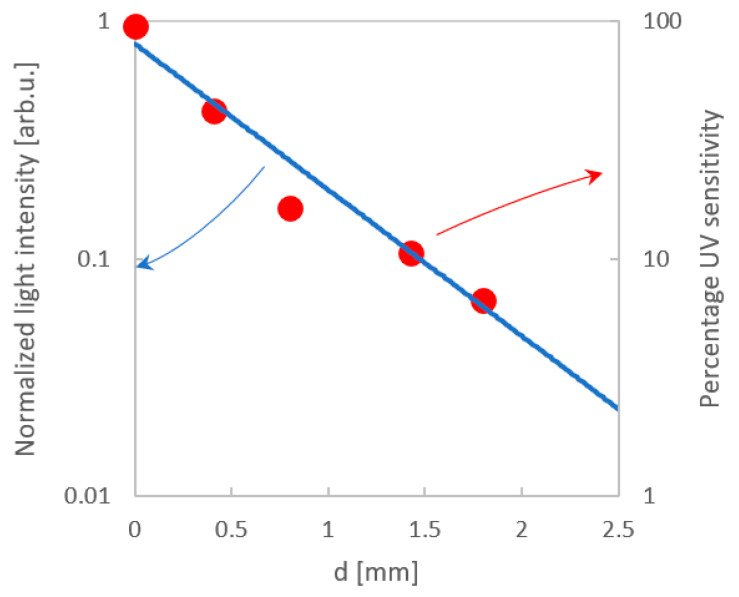
Comparison between percentage sensitivity of the laminated UV sensor (red dots) and the change in light intensity at a given plate depth (blue line).

When comparing the percentage change in sensitivity of the laminated UV sensor versus lamination depth to the change in light intensity presented in Figure 10, it can be seen that these two relationships are consistent. It follows that the change in sensitivity to UV radiation results directly from the attenuation of the composite material and the sensors were not damaged during the lamination process.

## 4. Summary

The presented work shows that it is possible to laminate a fiber optic UV sensor using FBG in a composite material. However, to protect the GO layer on the surface of the optical fiber, it is necessary to cover the GO layer with a thin layer of PMMA. As demonstrated by the tests, the PMMA layer does not adversely affect the UV sensor. What is more, the protective layer allows to significantly increase the sensor’s operating range.

The presented UV sensor allows to measure UV radiation both on the surface of the composite material and inside of it. For use in real measurement systems, however, both temperature and deformation must be monitored to distinguish them from changes in the measured UV light intensity. Thanks to this, it may be possible in future to link the intensity of UV radiation falling on the composite material with its degradation under the influence of this radiation. On the other hand, the presented sensor can be used to monitor UV radiation during the lamination process of light-curing materials. However, both solutions require further research.

## Figures and Tables

**Table 1:** Comparison of safe use of a fiber optic UV sensor before and after coating with a UV protective layer.

Sample Type	GO	GO + PMMA
Light intensity [mW/cm^2^]	160	500
